# Event history analysis of the duration of online public opinions regarding major health emergencies

**DOI:** 10.3389/fpsyg.2022.954559

**Published:** 2022-09-13

**Authors:** Xiaoyan Liu, Jiarui Zhao, Ran Liu, Kai Liu

**Affiliations:** ^1^School of Languages and Communication Studies, Beijing Jiaotong University, Beijing, China; ^2^School of Management and Economics, Beijing Institute of Technology, Beijing, China

**Keywords:** event history analysis, health emergencies, online public opinions, information lifecycle, emergency management

## Abstract

Based on event history analysis, this study examined the survival distribution of the duration of online public opinions related to major health emergencies and its influencing factors. We analyzed the data of such emergencies (*N* = 125) that took place in China during a period of 10 years (2012–2021). The results of the Kaplan-Meier method and Cox proportional hazards regression analysis showed that the average duration of online public opinions regarding health emergencies is 43 days, and the median is 19 days, which dispels the myth of the “Seven-day Law of Propagation.” Furthermore, the duration of online public opinions can be divided into three stages: the rapid decline stage (0–50 days), the slowdown stage (51–200 days), and the disappearing stage (after 200 days). In addition, the type of event, and the volume of both social media discussion and traditional media coverage all had significant impacts on the duration. Our findings provide practical implications for the carrying out of targeted and stage-based governance of public opinions.

## Introduction

The impacts of health emergencies can be generally measured and evaluated based on their spread, the extent of the impact, and their duration. The duration refers to the influential lasting time of health emergencies on social media, and reflects the degree of public attention on the events and while also reflecting the importance of the public health emergency. It also reflects the degree of severity of online public opinions (Lian et al., 2017; Dong et al., 2018). Typically, the longer the duration of a health emergency, the more attention it arouses and the greater the impact it asserts.

Duration is an important variable in the study of public opinions online regarding public health emergencies, and is one of the important mechanisms in terms of the evolution of online public opinion, which can affect both predictions and decision-making by governments, enterprises, and organizations, who often hope that online opinions dissipate relatively quickly–as short as possible– so as to minimize its negative impacts.

The “Seven-day Law of Propagation” is a popular idea when it comes to information on the Internet. It refers to the belief that the life span of emerging events online is only 7 days (Zhang, 2019).

Kwak et al. (2010) used data from Twitter and concluded that the active period of any widespread public opinion lasts no more than a week, and with 31% of the lifespans found lasting for only one day.

This paper argues the assertion of ideas of duration is more a summary of personal experience, or just a subjective guess, lacking empirical data support and scientific rigor. The prevalence of such so-called laws instead cause misunderstandings which can impact the governance of public opinions by government officers, and can lead to improper judgments and wrongful management practices.

Kwak’s study of duration focused on Twitter, not on public health events. Furthermore, the findings have yet to be verified on other social media platforms.

The detection and tracking of emerging hot topics or rumors on social media has been studied over the course of decades (Allan et al., 1998; Zheng and Li, 2009; Lu et al., 2013), and better algorithm explored is being applied to enhance its accuracy and efficiency (Yang et al., 2015; Alkhodair et al., 2020). There has been research done into the speed, scale, and scope of information propagation (Yang and Counts, 2010). However, current research on the duration of public opinion is very sparse.

Duration is a quantitative indicator and measurement of the life cycle of information. Defining the life cycle of public opinions regarding emergencies is derived from the concept of the life cycle of living things. Public opinions of emergencies have a beginning point as well as an end point where they are no longer discussed, and the evolution from start to finish appears to follow certain laws. From the emergence of a public opinion, to its stabilization and eventual extinction, public opinion can be divided into different stages, with Fink (1986) proposing four stages to the evolution of crisis communication in their theoretical framework.

Borrowing from the concept of the biological life cycle, American scholar Horton proposed the concept of an information lifecycle similar to the cyclical pathway of life, proposing four stages: birth, growth, decay, and death. Later scholars have proposed different online public opinion stage divisions, including a three-stage division (Zeng et al., 2014; Wang et al., 2020; Li and Shen, 2021), a four-stage division (Gu et al., 2014), a five-stage division (Xie et al., 2010), and finally a six-stage division (Zeng et al., 2014). However, many of these models are based on a subjective guess or qualitative perspectives, lacking empirical data. Even in the empirical studies, only a handful of cases were applied, while still lacking exact time divisions and a determination of the survival distribution of the duration.

Research on the life cycle of online public opinions not only reflects the evolutionary law of online public opinion, but also requires corresponding countermeasures at different stages of public opinions evolution. When an event receives less coverage or discussion both on- and offline, its impacts die away. Thus, because of the differences in their degrees of importance, public opinions about emergencies can have a variety of different life spans. For important emergencies, opinions’ life spans could last for weeks, while for flash events, they might die out in just a few days (Kleinberg, 2003).

The theory of the information lifecycle provides a theoretical framework to help understand the duration of online public opinions of health emergencies. The next section of this paper puts forth our research questions while summarizing past studies and identifying gaps in research.

The evolution of online public opinion involves primarily the functions of various factors such as the attributes of the event and its participants. Event attributes include classification and grading; participants refer to various social actors, including non-professional social media accounts coming from a variety of social roles, as well as professional individual opinion leaders (Zhang et al., 2021). At present, research on the duration of online public opinion regarding emergencies have focused on the attributes of the event itself, as well as other factors including media participation, opinion leaders, and death toll.

### Event attributes

Event attributes comprise classification and grading. The classification and grading of emergencies is one of the basic tasks for establishing an emergency management system (Yang et al., 2005). Xue and Zhong (2005) believe that different types of emergencies cause different situations of criticalities and social harms, and thus require different national emergency measures. Event type is an important independent variable when it comes to public opinion research. By crawling the data of five million YouTube user videos, Crane and Sornette (2008) divided social events into four different types: exogenous, endogenous, critical, and subcritical. Exogenous and endogenous refer to the type of disturbance, while critical and subcritical indicate the user’s ability to influence others into action. Additionally, they created combinations of the four types, then classified into four popularity patterns: exogenous subcritical, exogenous critical, endogenous critical, and endogenous subcritical. Utilizing the patterns, Kwak et al. (2010) studied the duration of these four patterns on Twitter. Fujita et al. (2018) estimated the influence of exogenous and endogenous forces on events. Other studies have found that there are significant differences in the survival times of three types of incidents: terrorist attacks, mass incidents, and criminal cases (Chen and Li, 2016). Meanwhile, Chen (2014) analyzed the survival distribution of public opinion in health events, disasters, and emergencies according to the different event types.

### Media participation

Media participation considers both traditional media and social media. Media can have positive or negative impacts by affecting personal emotions and views (Ma et al., 2014). In times of emergencies, media is an important channel for the public to obtain information. Information spreads quickly through the media and can work to alleviate people’s anxiety. In China, traditional media such as newspapers, radio, and television are still the primary media channels trusted by the public particularly when emergencies occur. Especially in these emergency cases, traditional media is perceived as being more authoritative. Compared to traditional media, social media have its own advantages and unique characteristics. When traditional media is unavailable, social media can serve as an important information channel in crises and emergencies (Macias et al., 2009) offering alternative views (Zhang et al., 2021). During emergencies, while social media has in the past been utilized by the general public to communicate, it is now becoming adopted by emergency responders, governments, and non-governmental organizations as an integral tool for disaster management (Simon et al., 2015). Social media accounts offer an opportunity to rapidly distribute critical information and, in doing so, to mitigate the impact of emergencies by influencing public reactions (Panagiotopoulos et al., 2016).

As of December 2021, the number of Internet users in China had reached 1.032 billion, and the Internet penetration rate had reached 73 percent (China Internet Network Information Center, 2022). Social media provides spaces for both officials and average citizens to seek to interpret emergency situations and intervene accordingly. Some studies have pointed out that active participation on social media prolongs the discussion time of network events.

### Opinion leaders

Lazarsfeld and Katz (1955) formulated a breakthrough theory of public opinion formation that sought to reconcile the role of media influence with the growing realization that, in a variety of decision-making scenarios ranging from political to personal, individuals may be influenced more by opinion leaders than media. Opinion leaders have a large number of followers and loud voices (Ekmen and Altın-Kayhan, 2017), giving them a stronger ability to spread public health opinions (Zhao et al., 2022). They usually strengthen microblog users’ subjective evaluations of events (Su, 2019), and thus have great influence on public sentiment (Zhao et al., 2014). This is more likely to trigger the emotional responses and emotional resonance/empathy of online groups (D’Ancona, 2017). Gao (2017) used survival analysis to conduct research on Weibo, and found that the number of opinion leaders voicing their thoughts has a significant impact on the duration of public opinions online. Li and Shen (2021) found that key nodes play important roles in spreading public opinion of animal epidemic emergency.

### Number of deaths

The more deaths caused by an event, the more attention it will receive on social media, and the longer the duration of public opinions regarding the event (Duan et al., 2020). Thus, this is an important factor in the event rating and is also the focus of public concern.

In this paper, we selected public health emergencies within China as samples to analyze the distribution of the duration of public opinions, and further explore their influencing factors.

## Research questions

The information life cycle provides a theoretical basis for the duration of public opinion, but this is only a speculative assumption lacking empirical findings when it comes to public opinions regarding health emergencies. As to the limited studies done examining Twitter, validation studies on other social media platforms are also needed to confirm the Twitter findings. All in all, there are currently very few empirical studies on duration. Therefore, while the information life cycle provides us with a theoretical framework, there is a lack of empirical research on duration. The present study then posed the two following research questions:

Research Question 1(RQ 1): What is the survival distribution of duration of online public opinions regarding health emergencies?

Research Question 2(RQ 2): What are the influencing factors of duration?

## Materials and methods

### Event history analysis

Event history analysis is a statistical method used to analyze the occurrence and timing of events within a given time, allowing some cases to be censored.

Event history analysis has different terminology in different disciplines. In sociology, it is called history analysis, such as the duration of a relationship from marriage to divorce, from unemployment to re-employment, etc. In medicine, it is called survival analysis, defining, for example, a patient’s survival time after the onset of a certain disease. In the field of engineering, it is called either reliability analysis and failure time analysis. Economics refers to duration analysis and transition analysis (Allison, 2010). Despite these differences in terminology, however, the concept is identical across disciplines. The current research analyzes the duration of public opinions regarding health emergencies (events), so we chose the term “event history analysis.”

The focus of event history analysis is to determine a specific model that characterizes the survival distribution, and to make statistical inferences based on the model. Event history analysis can be used to solve two problems that traditional multivariate statistical methods cannot solve: censoring and time-dependent covariates (Allison, 2010). A hazard model is able to handle censored observations containing partial information and covariates that change dynamically during the observing period. These two distinguishing features differentiate it from other regression models (Vermunt, 2001).

#### Definition of key concepts

Event refers to the end point of an emergency. The outbreak of public opinions on social media regarding public emergencies is considered the starting point of an event, and the cessation of public opinions is called the end point.

The outbreak of an event refers to the appearance of a topic in a document stream which is signaled by a “burst of activity,” with certain features rising sharply in frequency as the topic emerges (Kleinberg, 2003).

Duration is the time interval between the outbreak of an event and its end point, namely, the lasting time of online public opinions, which in this study is measured by days.

Censoring is a universal feature of event history analysis, with the most basic distinction being left censoring and right censoring (Allison, 2010).

#### The Kaplan-Meier method

The Kaplan-Meier (KM) estimator, also known as the product-limit estimator, is most widely used for estimating survivor functions rather than for demonstrating correlations. The cumulative distribution function estimates for the Kaplan-Meier model are as follows:


$$\hat{S}\,\left(t\right)=\left\{ \begin{array}{c} 1,t<t_{1}\\ \prod_{t_{i}\ll t}\left\{1-\frac{d_{i}}{y_{i}}\right\},t_{1}<t \end{array}\right.$$

$$\hat{V}\,\left[\hat{s}\,\left(t\right)\right]={\left[\hat{s}\,\left(t\right)\right]}^{2}\,\sum_{t_{i}}\frac{d_{i}}{y_{i}\,\left(y_{i}-d_{i}\right)}$$

*t*_*i*_ is the time point when the *i*_*th*_ event occurs, *d*_*i*_ is the number of events that occurred at time *t*_*i*_, *y*_*i*_ is the number of risks at time *t*_*i*_, and$\hat{V}\,a\,r\,\left[\hat{s}\,\left(t\right)\right]$is the variance estimation of the survival/persistence rate.

We also use the Log Rank test and Breslow to test the difference of the duration between and among the independent variables.

#### The Cox proportional hazards model

The semi-parametric proportional hazards model proposed by the British statistician D. R. Cox in 1972 is referred to as the Cox proportional-hazards model, the dependent variable of which is the hazard function. The Cox proportional-hazards model does not directly reflect the relationship between the survival function and the explanatory variables*X*_1_, *X*_2_,…*X*_*p*_, but uses the hazard function Inh(*t*) as the dependent variable. The regression coefficient β_*p*_ reflects exp (β_*p*_), the change in the risk ratio caused by per-unit change of *X*_*p*_ when other independent variables are fixed. Our study uses this model to evaluate the online influence of independent variables on the risk rate of deaths in public events. This model can be written as follows:


$$\left[h\,\left(t,X_{i}\right)=h_{0}\,\left(t\right)\,e\,x\,p\,\left(\beta_{1}\,X_{1}+\beta_{2}\,X_{2}+\cdots \,\beta_{p}\,X_{p}\right)\right]$$

*X*_1_,*X*_2_,… *X*_*p*_ are risk factors, which are the related factors that affect survival time. Estimating from the sample, β_1_, β_2, …_ β_*p*_ are regression coefficients. *h*_0_(*t*) is the baseline hazard and represents the hazard when all of the predictors and independent variables are equal to zero. If β_1_ is greater than 0, it indicates that the covariate is a risk factor. The higher the value, the shorter the survival time. If β_1_ is less than 0, it indicates that the covariate is a protective factor.

### Data collection

To keep our samples authoritative, we searched key words under “health emergency” and “public health event” at the official websites for the national and provincial Health Commissions, the Ministry of Emergency Management, and the provincial Emergency Management Departments of China as well as a global web search conducted on other search sites. Following the classification criteria of health emergencies according to the National Emergency Plan for Public Health Emergencies, we screened events deemed major (Grade III), significant (Grade II), and extremely significant (Grade I) that occurred between 2012 and 2021, and obtained a total of 125 events. We then entered the keywords for each event into the Sina Weibo platform and data collection was conducted by a web crawler in Python.

### Dependent variable: Duration

Sina Weibo (hereafter Weibo) is a Chinese version of Twitter, launched by Sina Corporation in 2009. As one of the leading and the most popular social media platforms in China, Weibo had 573 million monthly active users in March 2021 (NASDAQ, 2022). Referring to the operational definition of Zhao (2017) for event duration, the earliest posting time on Weibo was recorded as the starting time, the total number of posts on the earliest day recorded as *N*_0_, and the highest daily posting volume recorded as the peak *N*_1_. The death of the event was recorded as when the daily posting volume dropped to 10% of *N*_1_. Therefore, duration refers to the lasting time for the number of posts to drop from *N*_0_ to 10% of *N*_1_ (Zhao et al., 2017).

### Independent variables

#### Event types

According to the Regulations on Preparedness for and Response to Emergent Public Health Hazards, public health emergencies refers to “the sudden outbreaks of major infectious diseases, mass diseases of unknown cause, major food and occupational poisoning, and other events that seriously affect public health and cause or may cause serious damage to public health.”

This paper classifies health emergencies into the following five categories according to their nature with reference to the classification of types of health emergencies listed in the Regulation on Responses to Public Health Emergencies formulated and promulgated by The State Council, Grading Standards for Public Health Emergencies, issued by The Chinese Center for Disease Control and Prevention.

##### Food safety accidents

According to the Food Safety Law of the People’s Republic of China, a food safety accidents refers to “food-borne diseases, food contamination, and other food-derived accidents that are or may be harmful to human health” (The State Council of the People’s Republic of China, 2020).

##### Occupational poisoning accidents

This refers to workers coming into contact with industrial poisons in the process of labor, which may result in likely multiple organ damage.

##### Infectious diseases

Infectious diseases are defined by the Chinese Center for Disease Control and Prevention as diseases caused by various pathogens that can be transmitted from person to person, animal to animal, or human to animal.

##### Environmental pollution

This refers primarily to sudden events caused by natural disasters or initiated by man-made factors that have destroyed or damaged the environment such that it then endangers human health.

##### Medicine and health care accidents

Such accidents may occur throughout the entire production and sales process of medical instruments and drugs. They may be caused by the failure of the parties responsible for complying with relevant national laws and regulations in the selection of raw materials and manufacturing process of the drugs, or the great potential safety hazard in the production process, in either case resulting in the production of drugs that do not meet national standards, and cause great harm to human health and negative impacts on society.

#### Covariates

We make a tentative claim that the following eight variables might affect the duration and distribution of health emergencies: social media discussion volume, coverage volume by traditional media, participation of opinion leaders, subject of liability, areas influenced, number of people involved, and size of the city where the incident occurs. See Table 1 for coding of those independent and dependent variables.

**TABLE 1:** Variable coding.

Variable	Meanings	Coding
X_1_	Event type	1. Food safety; 2. Occupational poisoning; 3. Infectious disease; 4. Environmental pollution; 5. Medicine and health
X_2_	Social media discussion volume	Continuous variable
X_3_	Traditional media coverage	1. 0; 2. 1–5; 3. 6–50; 4. 51–1,000; 5 More than 1,000
X_4_	Opinion leader	1. Yes; 0. No
X_5_	Subject of liability	1. Government; 2. Schools; 3. Enterprises; 4. Individual; 5. Mixed
X_6_	Influenced areas	1. Single city and county; 2. Multiple cities; 3. Multiple provinces
X_7_	Number of people involved	1. Extremely large; 2. Large; 3. Medium; 4. Small
X_8_	City size	1. Extra large; 2. Large; 3. Medium; 4. Small
Time	Duration	Continuous variable
Event	Status	1. Death; 0. Censoring

##### Social media discussion volume

In this paper, the number of posts on relevant topics on Weibo has been selected as an indicator of social media discussion volume, a continuous variable, to measure the amount of discussion taking place on social media.

##### Traditional media coverage

We used WiseSearch as the data source, and analyzed the reports of news outlets found in the database to determine whether traditional media coverage can have an impact on public health emergencies. This independent variable is treated as categorical variable according to its coverage and divided into five groups: no coverage; 1 to 5 coverage; 6 to 50 coverage; 51–1,000 coverage; and coverage of more than 10,000.

##### Participation of opinion leaders

Opinion leaders on Weibo are influencer users, and are identified publicly as verified users known as “Big Vs,” denoted by a verification badge, a capitalized letter “V” added alongside their account name (Wang et al., 2014). In this paper, the “Big Vs” on Weibo (with more than 500,000 followers) are taken as the dichotomous variable.

##### Subject of liability

This refers to the main body chiefly responsible for public health emergencies. It is divided into five categories: government, school, enterprise, individual, and mixed.

##### Influenced areas

This refers to the geographical areas affected by a particular public health emergency. According to the classification of health emergencies in the Regulations on Emergency Response to Public, Health Emergencies issued by the State Council of the People’s Republic of China, the influenced scope of incidents falls into three tiers: a single city or county, multiple cities, and multiple provinces.

##### Number of people involved

This refers to the number of people affected by a health emergency, including both those who have died and those who are injured. According to the National Emergency Plan for Public Health Emergencies, the number of casualties are classified into four categories: extremely large (501and above), large (101 to 500), medium (31 to 100), and small (1 to 30).

##### Size of the city where the public health emergency occurs

This is usually rated by the number of its residents. Generally speaking, larger cities tend to attract more media and public attention. According to the Notice of the State Council on Adjusting the Standards for Categorizing City Sizes, cities in Mainland China are divided into: extremely large cities (a permanent urban population of more than 2 million), large cities (a permanent urban population of between 500,000 and 1 million), medium cities (a permanent urban resident population of between 200,000 and 500,000), and small cities (a permanent urban population of less than 200,000) (The State Council of the People’s Republic of China, 2014).

## Results

### Duration of online public opinions regarding major health emergencies

The sample (*N* = 125) comprised 30 cases of food safety accidents, 31 cases of occupational poisoning, 42 cases of infectious diseases, 12 cases of environmental pollution accidents, and 10 cases of medical and health accidents. Meanwhile, six cases were censored, accounting for 4.8% of the total cases, as shown in Table 2.

**TABLE 2:** Proportion of each event type.

Event type	Total	Number of events	Censored	
			*N*	Percent
Food safety	30	30	0	0.0%
Occupational poisoning	31	28	3	9.7%
Infectious disease	42	39	3	7.1%
Environmental pollution	12	12	0	0.0%
Medicine and health	10	10	0	0.0%
Total	125	119	6	4.8%

### Univariate analysis of duration

Overall, 32% of the online public opinions regarding health emergencies lasted for less than 7 days, 39.50% for 8–50 days, 12.61% for 51–100 days, 6.72% for 101–150 days, 3.36% for 151–200 days, and 9.24% for more than 200 days (see Table 3).

**TABLE 3:** Duration distribution.

Lifetime	Frequency number	Frequency (%)
0–7 days	40	32.00%
8–50 days	47	39.50%
51–100 days	15	12.61%
101–50 days	8	6.72%
151–200 days	4	3.36%
More than 200 days	11	9.24%

The overall mean of duration of public opinions was 43.50 days. The medical and health events topped the list with 99.30 days each, followed by environmental pollution events with 64.67 days. The duration of occupational poisoning cases and infectious disease events was 45.71and 39.74 days, respectively. Food safety incidents showed an average duration of 19.26 days, the shortest of all five categories. The median of the five types of events was14, 13, 19, 36, and 65 days, respectively, as shown in Table 4.

**TABLE 4:** Descriptive statistics of event type.

Event type	Mean				Median			
	Estimate	Std. error	95% Confidence interval		Estimate	Std. error	95% Confidence interval	
			Lower bound	Upper bound			Lower bound	Upper bound
Food safety	19.267	3.662	12.090	26.443	14.000	4.782	4.627	23.373
Occupational poisoning	45.714	11.888	22.413	69.016	13.000	5.953	1.332	24.668
Infectious disease	39.744	9.039	22.027	57.460	19.000	3.113	12.898	25.102
Environmental pollution	64.667	17.962	29.461	99.873	36.000	19.053	0.000	73.343
Medicine and health	99.300	32.248	36.093	162.507	65.000	43.481	0.000	150.223
Overall	43.504	5.538	32.650	54.358	19.000	3.354	12.427	25.573

Unit = day.

Kaplan-Meier analysis of the influencing factors can estimate the survival functions of duration and demonstrate whether there is correlation between the different independent variables and duration. Log Rank test results showed that there is significant difference of duration between or among event types (*p* = 0.012 < 0.05). Log Rank test results also showed that traditional media coverage (*p* = 0.019 < 0.05) and city size (*p* = 0.032 < 0.05) had significant impacts on duration. Breslow test results showed that influenced areas had a marginal significant impact on duration (*p* = 0.10 < 0.1). Other variables, including subject of liability, number of people involved, and opinion leaders, were not significant, so we put all the independent variables into the Cox model for further analysis, as shown in Table 5.

**TABLE 5:** Log Rank and Breslow tests.

	Log Rank (Mantel-Cox)			Breslow (Generalized Wilcoxon)		
	Chi-square	*df*	Sig.	Chi-square	*df*	Sig.
Event type	12.828	4	0.012	6.045	4	0.196
Traditional media coverage	11.741	4	0.019	16.493	4	0.002
City size	8.834	3	0.032	8.484	3	0.037
Influenced areas	1.862	2	0.394	4.612	2	0.100
Subject of liability	4.793	4	0.309	2.947	4	0.567
Number of people involved	2.546	3	0.467	1.92	3	0.589
Opinion leaders	1.907	1	0.167	2.136	1	0.144

### Cox model results analysis

#### The three stages of duration

**FIGURE 1:**
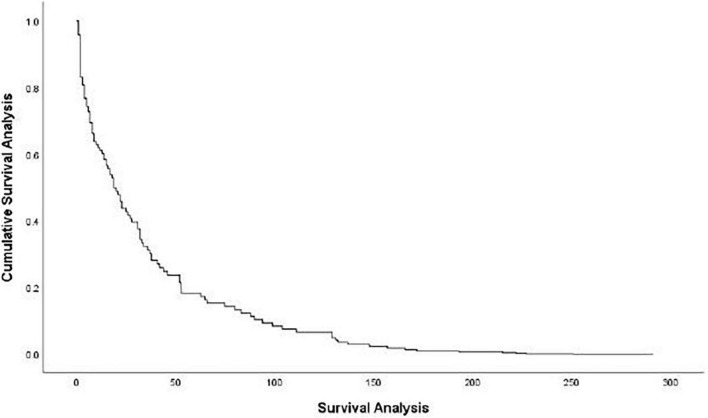
Survival function at mean of covariates.

The three stages of duration can be outlined as follows: the first stage (0–50 days) is featured by a rapidly descending rate of survival function. It levels off at the second stage (51–200 days). At the third stage (more than 200 days after the event) the impacts of the events basically subsided (see Figure 1).

According to the results of the Omnibus Tests of model coefficients, –2 Log Likelihood = 870.784, and *p* = 0.010.

As we can see in the Cox model, event type and media participation including social media discussion volume and traditional media coverage have a significant effect on the observed duration of public opinions online.

#### Effect of event type

**FIGURE 2:**
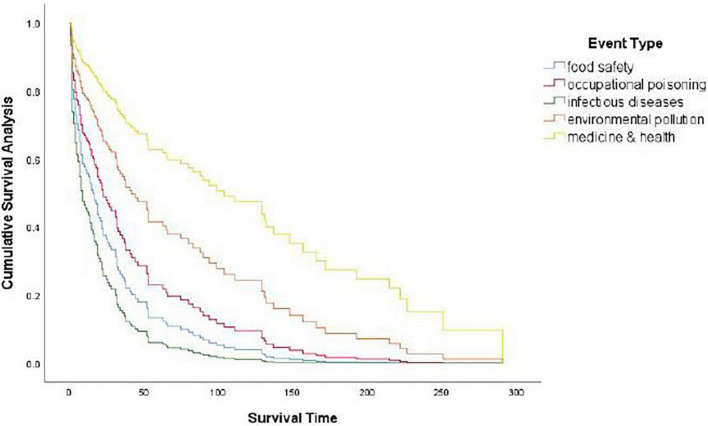
Survival function of event types.

With other variables under control, at the 95% confidence level there were significant differences on the duration of event types (*p* = 0.037 < 0.05). Compared with the medical and health events group, the duration risk of food safety events increased by 332.1%, the duration risk of occupational poisoning events increased by 215.4%, and the duration risk of deadly infectious diseases increased by 499.6%. Meanwhile, the difference between medical and health events and environmental pollution was not found to be significant on duration risk (see Table 6 and Figure 2).

**TABLE 6:** Multivariate Cox regression analysis results.

Variable	B	*SE*	Wald	*df*	Sig.	Exp (β)	95.0% CI for Exp (β)	
							Lower	Upper
**Event type**								
Medical and health (=0)			10.197	4	0.037			
Food safety	1.463	0.517	8.001	1	0.005	4.321	1.567	11.910
Occupational poisoning	1.149	0.567	4.110	1	0.043	3.154	1.039	9.574
Infectious disease	1.791	0.709	6.390	1	0.011	5.996	1.495	24.042
Environmental pollution	0.633	0.602	1.106	1	0.293	1.884	0.579	6.131
**Social media discussion volume**	−0.017	0.009	3.969	1	0.046	0.983	0.966	1.000
**Traditional media coverage**								
More than 1,000 (=0)			9.721	4	0.045			
0	1.057	0.457	5.364	1	0.021	2.879	1.177	7.043
1–5	1.018	0.481	4.471	1	0.034	2.767	1.077	7.106
6–50	0.422	0.471	0.803	1	0.370	1.525	0.606	3.835
51–1000	0.239	0.417	0.330	1	0.566	1.270	0.561	2.876
**Opinion leaders**								
No opinion leaders (= 0)	0.224	0.245	0.835	1	0.361	1.252	0.774	2.025
**Subject of liability**								
Mixed (=0)			2.147	4	0.709			
Government	−0.016	0.717	0.001	1	0.982	0.984	0.241	4.015
School	0.201	0.697	0.083	1	0.773	1.223	0.312	4.793
Enterprise	0.441	0.641	0.473	1	0.492	1.554	0.442	5.464
Individual	0.603	0.650	0.863	1	0.353	1.828	0.512	6.532
**Influenced areas**								
Multiple provinces (=0)			0.567	2	0.753			
Single city or county	−0.344	0.476	0.523	1	0.470	0.709	0.279	1.801
Multiple cities	−0.274	0.456	0.362	1	0.547	0.760	0.311	1.857
**Number of people involved**								
Small (1–30) (=0)			4.129	3	0.248			
Extremely large (501and above)	0.745	0.382	3.799	1	0.051	2.107	0.996	4.457
Large (101–500)	0.483	0.386	1.570	1	0.210	1.621	0.761	3.454
Medium (31–100)	0.501	0.375	1.788	1	0.181	1.651	0.792	3.443
**City size**								
Small city (=0)			4.000	3	0.261			
Extremely large city	−0.281	0.277	1.028	1	0.311	0.755	0.439	1.300
Large city	−0.879	0.501	3.079	1	0.079	0.415	0.156	1.108
Medium city	−0.470	0.309	2.319	1	0.128	0.625	0.341	1.145

#### Effect of media participation

With other variables controlled, at the 95% confidence level, social media discussion volume (*p* = 0.046 < 0.05) and traditional media coverage (*p* = 0.045 < 0.05) both had significant effects on duration.

For social media, the β value of discussion volume was −0.017, which means it is a protective factor with a relative duration risk of 0.983. With every unit increase in the amount of social media discussion, the duration risk decreased by 1.7% [(1–0.983) × 100%], which also meant that its probability of prolonged duration was increased by 1.7% with every unit increase.

For traditional media, compared with the reference group with the highest coverage, the duration risk of no coverage increased by 187.9%, while the duration risk of coverage (1–5) increased by 176.7%. The duration risk of the rest of the two groups did not show significant difference between the reference groups.

Other variables such as the participation of Opinion leaders, subject of liability, influenced areas, number of people involved, and city size did not seem to be important factors in the duration of public opinions.

## Discussion

Using event history analysis, this study examined the duration of online public opinions regarding major health emergencies in China occurring between 2012 and 2021. Results show that the mean duration of public opinion was 43 days, with a median of 19 days.

Online public opinions about major health emergencies follow a life cycle which runs through consecutive stages. In light of the law of public opinion evolution, our event history analysis revealed three stages. The first stage (0–50 days) is featured by a rapid decline in discussion, followed by a slowdown at the second stage (51–200 days), and finally the disappearance of impacts in the third stage (over 200 days).

Following our understanding of the survival distribution of duration, we analyzed the influencing factors and found that with other variables controlled and at the 95% confidence level, the variables of event type, media participation including social media discussion volume and traditional media coverage all had significant impacts on duration.

A Cox model showed that event type played the most important role in the duration of public opinions online. Of all the event types, food safety had the shortest duration with a mean of 19 days and a median of 14 days, while medicine and health lasted the longest time with an average of 99 days and a median of 65 days.

Media participation was shown to be an important factor in duration. Social media discussion volume and traditional media coverage both had significant effects on duration. With regards to social media, more discussion online increased the duration of public opinion.

Although opinion leaders do not show significant impact on duration in the Cox regression model, the variable nonetheless merits attention. Within our data, the β value of opinion leaderswas.224, and exp (β) was 1,252, which indicates that the duration risk of events with opinion leaders was increased by 25.2% compared to those with no engaged opinion leaders. Even though the number of people involved was not found to be significant, events with increased casualties also are deserving of particular attention.

We also explored the interactive effects of coverage of traditional media and discussion volume on social media in our model, however, the interactive term of coverage of traditional media discussion volume on social media was not significant (*p* = 0.73 > 0.05). The interactive term did not show significant impacts on duration of online public opinions. As the interactive effects did not exist, we excluded the interactive term in our model.

### Theoretical and practical implications

This paper has important theoretical and practical significance for the study of duration and its influencing factors. With the theory of the information life cycle as our theoretical framework, we undertook an empirical study on the duration of online public opinions examining 125 major health emergencies that took place in China from 2012 to 2021. The finding of the three-stage model of duration as developed from our empirical data further expands information life cycle theory. Given that the literature is still very limited at present, we feel that this is a substantial contribution to this field. Furthermore, considering that duration and its influencing factors are rarely researched at present and that consideration of its variables in research is far from systematic, this paper marks a bold attempt to explore the factors that influence public opinion duration.

With regards to the practical implications of our findings, first, our research helps dispel the longstanding misconception of the “Seven-day Law of Propagation.” Our research serves as a wake-up call for government and other officials who need to understand the duration patterns of online public opinions as they plan and manage public opinions in response to emergency events lasting for longer periods of time.

Events that continue over a long period of time can trigger discussion mechanisms of associated events. For example, online public opinion surrounding a medicine scandal that broke out in a nursery school in Xi’an, Shaanxi Province, was alive and active online for 291 days. Meanwhile, discussions surrounding the African Swine Fever epidemic in Mingshui, a county in China’s northernmost province of Heilongjiang, sustained online presence for 251 days. Public opinions of an event do eventually subside, however, other events may evoke collective memory and prompt further online discussion about other ongoing related events. Halbwachs (1992) sees collective memory as something shared by a group that has spiritual meaning. Some health emergencies have significant social impacts, and the recurrence of similar events tends to resonate with people and stimulate sustained discussions on social media.

Another example of public opinion leading to other tangential conversations is the “counterfeit vaccine.” In 2013, there was the production and sale of an extremely large amount of a poor-quality rabies vaccine for human use. The online public opinion it triggered subsided in 2 days, however, the online public opinions triggered by two other events, a substandard diphtheria vaccine in 2017 and a problematic Chang Sheng vaccine in 2018, lasted 65and 166 days, respectively. Posts showed that the current event revived discussions over the previous related vaccine events, with some netizens even retweeting previous reports, which pushed online public opinions to explode from just one single event into a major event over multiple problematic vaccines. The duration of online public opinion was thus prolonged.

Second, we have proposed a three-stage division of online public opinions on health events based on scientific empirical research, which sheds light on the value of targeted phased management of public opinion by government managers.

Third, there were significant differences found on the duration of different types of events (see Figure 2). Medical and health events had the longest duration time with a mean of 99.30 days, followed by environmental pollution events with 64.67 days, occupational poisoning and infectious disease events with 45.71 and 39.74 days, respectively, and food safety having the shortest mean duration at 19.26 days. The median of the five types of events was 65, 14, 13, 19, and 36 days, respectively (see Table 4).

The duration of medicine and health events was relatively long with a slow decline rate, which corresponds to the complex causes of such events and the requirement of ongoing follow-up reports. Though online public opinions regarding infectious disease events experience a fast drop at their early stages, there is a subsequent long flat period, as infectious diseases recur and spread easily, generating a continuous series of new but related topics in online public opinions (see Figure 2).

It is therefore necessary to follow the law of the public opinion duration for each specific event, with differentiated public opinion management procedures used for the various types of events. Governments should pay more attention to events with longer public opinion duration. On one hand, they should realize that it is common for public health events such as medicine and health or environmental pollution to have an online duration that lasts for a longer period of time. On the other hand, governments should publish information openly and transparently from the start (Ma et al., 2014) so that the unnecessary longer duration of public opinion can be minimized. Subjects involved should be clear and those responsible should make their voices heard at the earliest moment, as an effective way of mitigating online public opinions. We recommend that concerned governments take corresponding countermeasures against runaway public opinion in a timely manner, which can keep rumors from spreading and reduce unwanted discussions on social media platforms from the early stages of an event.

The fake milk powder event from 2020, in which several infants and children were reported to develop “big-head” disease and contracted rickets after drinking the powder sold by local pharmacies and baby stores in Chenzhou, Hunan Province. Fearing that their children would be left with chronic conditions, many parents wrote a joint open letter to the Chenzhou Mayor on March 30 asking for a thorough investigation of the case. Strong emotions were aroused and information spread rapidly across social media, while people’s memories of a similar event–the Sanlu Milk scandal of 2008 –were also recalled. This event sparked criticisms from netizens on social media, with people called for a thorough investigation into the case and tougher management over the formula industry. It wasn’t until May 13th at the State Administration for Market Regulation of China published a notification urging local authorities in Hunan Province to conduct a thorough investigation. On May14, the People’s Government of Hunan Province responded that local government would conduct a thorough investigation. Governments concerned about public opinion management should make full use of social media and respond immediately and effectively to prevent emotions going out of control or stopping the spread of rumors.

Fourth, media participation should be handled and managed properly. When public opinion was focused on violence toward medical staff in China, Duan et al. (2020) found that the more attention of the topic on Weibo, the more people participated in the discussion, and the longer the duration of public opinion. Our results also support this idea. Putting a focus on Weibo can be considered “discussion volume,” as at the 95% confidence level, social media discussion volume was significant and is a protective factor of duration risk, which shows that the more discussion volume on social media, the longer an event’s duration will last.

Traditional media, even now in the Internet Age, continues to have great social impact. The more traditional the media coverage, the more attention and interest it arouses in people, and the longer duration of public opinions shared online. Numerous reports may provide people with rich, detailed information, but they can also increase the uncertainty surrounding the event, leading to a continuous fermentation of public opinion. Furthermore, when serious consequences become the focus of traditional media attention, people’s perception of threat will grow, which does not help to soothe online public opinion. Therefore, traditional media as well as social media need to provide timely comprehensive analyses that share accurate and reliable information and increase people’s knowledge. At the same time, there is also need to control the quantity of traditional media coverage for the sake of enhancing quality. In addition to providing sufficient and accurate information to the public, media also need minimize redundant reporting lest such reports increase the public’s perceived threat and cause panic.

### Limitations

There are some limitations to this study. First, in terms of sample selection, given the absence of an authoritative database on public health emergencies, we derived our samples from extensive network search. Manual searches and subjective judgments may result in omissions of events, however. It is our hope that future researchers and relevant government agencies might initiate such a database to provide trustworthy data support for subsequent empirical studies. Second, there is room for discussion on the operational definition of time of death. This paper attempted to use the amount of reporting *N_0_* = *N*_1_, but it was found to be not feasible in actual data observation. Therefore, this paper refers to the defining method of Zhao (2017), and more scientific research can be carried out on the operational definition of time of death in subsequent studies. Third, public health emergencies are often complicated, that is, they are affected by multiple factors. In subsequent studies, we may study other possible variables and build effective models so as to discover further potential influencing factors. For instance, emotions are an important influencing factor on information diffusion on social media (Stieglitz and Dang-Xuan, 2013), where by the stronger netizens’ emotions, the stronger the diffusion of social media (Zeng and Zhu, 2019). Future studies could collect data including emotions and test their influence on duration. Fourth, this paper found the initial signs of the correlation between topics, but time and study design prevented further discussion. In the future, empirical research should be carried out on the rules of propagation and correlation among topics. Fifth, there may be a few exceptions of events that involved only a small number of people yet had a long duration, but our data indicated that the more discussion online, the longer the topic’s duration, which has also been previously supported by Duan et al. (2020). However, because of the limitations of the available data, we think this is worth considering in the future studies which may have access to more data. Finally, due to contextual constraints, we tried to explore the duration of online public opinions and its influencing factors, by focusing on 125 major health emergencies in China. The cases were only about China and examining Weibo. Future research could try to include more major health emergencies from across the globe, as well as explore data across other social media platforms.

## Data availability statement

The raw data supporting the conclusions of this article will be made available by the authors, without undue reservation.

## Author contributions

XL and JZ originated and designed the research. All authors contributed to the statistical analysis, interpretation of the results, revision of the manuscript, involved in editing, reviewing, providing feedback for this manuscript, and approved the final version to be published.
